# Anti-biofilm effects and healing promotion by silver oxynitrate-based dressings

**DOI:** 10.1038/s41598-022-26856-x

**Published:** 2023-02-03

**Authors:** Christopher Doherty, Charlotte V. Byrne, Sajwa Baqader, Cecile El-Chami, Andrew J. McBain, Helen A. Thomason

**Affiliations:** 1 Division of Pharmacy and Optometry, School of Health Sciences, Faculty of Biology, Medicine and Health, The University of Manchester, Manchester, M13 9PT UK; 2 3M Medical Solutions Division. King Edward Court, King Edward Road, Knutsford, Cheshire, WA16 0BE UK; 3 Community Nursing and Healthcare Department, Faculty of Nursing, Umm Al-Qura University, Mecca, Saudi Arabia; 4 School of Biological Sciences, Division of Musculoskeletal and Dermatological Sciences, Faculty of Biology, Medicine and Health, University of Manchester, Oxford Road, Manchester, M13 9PT UK

**Keywords:** Microbiology, Health care

## Abstract

Microbial growth within a wound often manifests as biofilms, which can prevent healing and is difficult to eradicate. Novel silver dressings claim to combat wound infection, but anti-biofilm efficacy and effects on healing independent of infection are often unclear. Using *in vitro* and *in vivo S. aureus* and *P. aeruginosa* biofilm models, we report the efficacy of a dressing which produces Ag^1+^ ions; an Ag^1+^ dressing containing ethylenediaminetetraacetic acid and benzethonium chloride (Ag^1+^/EDTA/BC), and a dressing containing silver oxynitrate (Ag Oxysalts) which produces Ag^1+^, Ag^2+^ and Ag^3+^ ions, against wound biofilms, and their effects on healing. Ag^1+^ dressings had minimal effect on *in vitro* and murine (C57BL/6j) wound biofilms. In contrast, Ag Oxysalts and Ag^1+^/EDTA/BC dressings significantly reduced viable bacteria within *in vitro* biofilms and demonstrated a visible reduction in bacteria and EPS components within murine wound biofilms. The dressings had different effects on the healing of biofilm-infected and uninfected wounds, with Ag Oxysalts dressings having a greater beneficial effect on re-epithelialisation, wound size and inflammation than the control treatment and the other silver dressings. The different physicochemical properties of the silver dressings result in varied effects on wound biofilms and healing which should be considered when selecting dressings to treat biofilm-infected wounds.

## Introduction

A chronic wound is defined as “a wound that has failed to proceed through the normal phases of wound healing in an orderly and timely manner”^1^. Chronic wounds present a psychological, social, and economic burden on patients and health care systems. The annual spend on wound care and associated comorbidities by the National Health Service in the UK was an estimated £8.3 billion in 2017/2018^2^. Chronic wounds are also currently a critical issue in the United States as Medicare estimates for treating wound care patients range from $28.1 billion to $96.8 billion per annum^3^.

Infection is a major contributing factor to the failure of a wound to heal. Infection often manifests as a biofilm with biofilms identified in 78% of non-healing chronic wounds. Biofilms occur when microorganisms irreversibly adhere to a surface such as the wound bed, and can aggregate to form a community producing extracellular polymeric substances (EPS). Wound biofilms have been associated with a heightened inflammatory response resulting in tissue damage which may delay or prevent healing^4^. Increases in tissue damage can be due in part to an increase in matrix metalloproteases, collagenase, elastase and reactive oxygen species activity^5^. Additionally, inflammatory cells and the biofilms themselves are high consumers of oxygen and thus may drive local tissue hypoxia, depleting cells of vital oxygen required for effective tissue repair^6^.

Mature biofilms are highly tolerant to antimicrobials and therefore an aggressive strategy is required to combat biofilm infection, such as mechanical debridement followed by potent antimicrobial therapies. As biofilms can reform quickly, effective antimicrobials reduce the risk of reformation following debridement^7^.

Silver is increasingly used in antimicrobial dressings and is often used as a first-line treatment for infected chronic wounds. There are a vast variety of commercially available silver dressings, each containing different silver formulations, concentrations, and base substrates. Advances in silver dressings have led to the development of novel silver dressings. Silver in its metallic form (Ag^0^) is inert; to gain antimicrobial efficacy it must lose an electron to form ionic silver (Ag^1+^). Traditional silver dressings contain silver compounds or metallic silver which when exposed to fluid breakdown to produce Ag^1+^ ions. These Ag^1+^ ions react with bacterial cells, removing electrons from structural components or key processes essential for survival. Propriety technology has led to the development of a novel silver compound, Ag Oxysalts (silver oxynitrate, Ag_7_NO_11_), which has been incorporated into wound dressings. Unlike traditional silvers, when Ag Oxysalts breakdown they produce higher valent states of silver (Ag^1+^, Ag^2+^ and Ag^3+^). In vitro studies have demonstrated that Ag Oxysalts are more effective at low concentrations than singly ionic silver (Ag^1+^) against pathogenic bacteria including *P. aeruginosa, S. aureus* and *E. coli*^8,9^. Another novel silver dressing has incorporated additional components, ethylenediaminetetraacetic acid (EDTA) and benzethonium chloride (BC) which reportedly target the biofilm EPS and thereby increase the penetration of silver into the biofilm. These novel silver technologies offer new methods of targeting wound biofilms. However, the effects these antimicrobials have on the wound environment and healing independent of infection are important to ensure they do not create a hostile wound environment and delay healing. Concerns over silver cytotoxicity have been reported for several silver dressings in vitro^10,11^. However in vitro cytotoxicity has not been translated to in vivo toxicity with several Ag^1+^ dressings expressing a good safety profile^12^.

Here we examine the efficacy of carboxymethyl cellulose dressings containing novel silver formulations against wound biofilms in vitro and in vivo. Furthermore, the impact of these dressings on the immune response and healing independent of infection is also assessed.

## Methods

### Dressings

All dressings used were commercially available. 3M Kerracel Gelling Fibre Dressing (3M, Knutsford, UK) is a non-antibacterial 100% carboxymethyl cellulose (CMC) gelling fibre dressing and was used as a control dressing in this study. Three antimicrobial silver CMC dressings were evaluated, 3M Kerracel Ag Dressing (3M, Knutsford, UK) which contains 1.7% w/w Ag Oxysalts (Ag_7_NO_11_) which have higher valent states (Ag^1+^, Ag^2+^ and Ag^3+^) silver ions. The Ag^1+^, Ag^2+^ and Ag^3+^ ions are produced in a ratio of 1:2:4 during the breakdown of Ag_7_NO_11_. Aquacel Ag Extra a dressing containing 1.2% silver chloride (Ag^1+^) (ConvaTec, Deeside, UK)^13^ and Aquacel Ag + Extra a dressing containing 1.2% silver chloride (Ag^1+^), EDTA and benzethonium chloride (ConvaTec, Deeside, UK)^14^.

### Bacteria

*Pseudomonas aeruginosa* NCTC strain 10781 (Public Health England, Salisbury, UK) and *S. aureus* NCTC 6571 (Public Health England, Salisbury, UK) were used in this study.

### Colony biofilm assays

Bacteria were incubated overnight in Mueller-Hinton broth (Oxoid, Altrincham, UK). The overnight culture was then diluted 1:100 in Mueller–Hinton broth and 200 µl applied to sterile Whatman cyclopore circular membrane 0.2 µm (Whatman plc, Maidstone, UK) placed on Mueller-Hinton agar plates (Sigma-Aldrich Company Ltd, Kent, UK) at 37 °C for 24 h to form colony biofilms. Log reduction testing was performed on these colony biofilms.

Dressings were cut into 3cm^2^ square pieces and pre-moistened with sterile deionised water. Dressings were placed over colony biofilms on agar plates. Every 24 h a biofilm was removed, and quantification of viable bacteria within the biofilm (CFU/ml) was calculated by vortexing the biofilm with sterile 3 mm glass beads (Merck-Millipore, Watford, UK) in Dey-Engley neutralising broth (Merck-Millipore), before serial diluting (10^–1^ to 10^–7^). Standard plate counts were performed on Mueller-Hinton agar following 24 h incubation at 37 °C. Each treatment and time-point were repeated in triplicate and replicate plate counts for each dilution were performed.

### Porcine ex vivo biofilm model

Porcine abdominal skin was obtained from female large white pigs within 15 min after being culled following European EEC export standards. The skin was shaved and cleaned with alcohol wipes before freezing at -80 °C for 24 h to render the skin non-viable. After defrosting, 1cm^2^ pieces of skin were washed three times in PBS, 0.6 % sodium hypochlorite, 70 % ethanol for 20 min each. Residual ethanol was removed by washing three times in sterile PBS before removing the epidermis. The skin was cultured on a stack of three absorbent pads (Merck-Millipore) topped with a 0.45 µm nylon membrane (Merck-Millipore) in a 6-well plate containing 3 mL Dulbecco's Modified Eagle Medium supplemented with 10% foetal calf serum (Sigma-Aldrich Company Ltd).

A colony biofilm was grown as described for the biofilm contact study. After culturing the biofilm on membranes for 72 h, the biofilm was applied to the dermal skin surface using a sterile inoculation loop and the membrane removed. The biofilm and was then cultured on the porcine dermal layer for a further 24 h at 37 °C to allow biofilm maturation and attachment to the porcine skin. Following biofilm maturation and attachment, 1.5cm^2^ pieces of dressing pre-moistened in sterile distilled H_2_O, were applied directly to the surface of the skin and incubated for 24 h at 37 °C. Viable bacteria were visualised via staining with PrestoBlue Cell Viability Reagent (Invitrogen, Life Technologies, Paisley, UK) applied evenly to the apical surface of each explant and incubated for 5 min. Images were captured immediately on a Leica MZ8 microscope using a Leica DFC425 digital camera. Areas of pink staining were quantified using Image Pro version 10 software (Media Cybernetics Inc, Rockville, MD Image-Pro (mediacy.com)). Scanning electron microscopy was performed as described below.

### Biofilm contact study

An overnight culture of bacteria was diluted 1:100 in Mueller–Hinton broth. 200 µl of culture was added onto a sterile Whatman cyclopore circular membrane 0.2 µm (Whatman, Maidstone, UK) and placed on Mueller-Hinton agar. Biofilm plates were incubated for 72 h at 37 °C to allow the formation of a mature biofilm.

After the biofilm had matured for 3 days, 3 cm^2^ square dressings were placed directly onto biofilms and incubated for 24 h at 37 °C. Following removal of dressings from the biofilm surface, 1 ml of PrestoBlue Cell Viability Reagent (Invitrogen, Waltham, MA) was added to the surface of each biofilm for 20 s. The surface was drained before recording the colour changes using a Nikon D2300 digital camera (Nikon UK Limited, Kingston, UK).

### Biofilm preparation for in vivo studies

An overnight culture was prepared in Mueller-Hinton agar and a single colony transferred to 10 ml Mueller-Hinton broth and incubated in a shaking incubator at 37° C (100 rpm). Following incubation overnight, the culture was diluted 1:100 in Mueller-Hinton broth and 300 µl applied to Whatman cyclopore circular membranes 0.2 µm (Whatman International, Maidstone, UK) placed on Mueller Hinton agar and incubated at 37° C for 72 h. Mature biofilms were applied to the wound as described below.

### Murine biofilm wound model

All animal work was carried out at the University of Manchester and performed under a project license approved by the institutional Animal Welfare and Ethical Review Body (P8721BD27) and according to the regulations issued by the Home Office under amended ASPA, 2012. All authors complied with ARRIVE guidelines. Eight-week-old C57BL/6j mice (Envigo, Oxon, UK) were used for all in vivo studies. Mice were anaesthetised with isoflurane (Piramal Critical Care Ltd, West Drayton, UK) and the dorsal surface was shaved and cleansed. Each mouse then received 2 × 6 mm excisional wounds using a Stiefel biopsy punch (Schuco International, Hertfordshire, UK). For biofilm-infected wounds, immediately after wounding, a 72 h colony biofilm, grown on membranes as described above, was applied to the dermal layer of the wound using a sterile inoculation loop, and the membrane discarded. One-centimetre square dressings were pre-moistened with sterile water to maintain a moist wound environment. Dressings were applied directly to each wound and covered with 3M Tegaderm Film (3M, Bracknell, UK), and Mastisol Liquid Adhesive (Eloquest Healthcare, Ferndale, MI) was applied to the edges to provide additional adherence. Buprenorphine (Animalcare, York, UK) was administered as analgesia at a concentration of 0.1 mg/kg. Mice were culled three days post-wounding using Schedule 1 method and the wound area was removed, bisected, and stored as required.

### Hematoxylin and eosin staining

Hematoxylin (ThermoFisher Scientific) and Eosin (ThermoFisher Scientific) staining was performed according to the manufacturers protocol. Wound area and re-epithelialisation were quantified using Image Pro version 10 software (Media Cybernetics Inc, Rockville, MD).

### Neutrophil and macrophage quantification

Tissue sections were deparaffinised in xylene (ThermoFisher Scientific, Loughborough, UK) and re-hydrated through a graded ethanol series of 100–50 % and briefly submerged in deionised water (ThermoFisher Scientific). Immunohistochemistry was performed using a VectaStain Elite ABC kit PK-6104 (Vector Laboratories, Burlingame, CA) according to the manufacturer’s protocol. Primary neutrophil NIMP-R14 (Thermofisher Scientific) and macrophage Ms CD107b Pure M3/84 (BD Biosciences, Wokingham, UK) antibodies were diluted 1:100 in blocking solution added to the section surface followed by secondary antibodies, VectaStain ABC and Vector Nova Red Peroxidase (HRP) Substrate Kit (Vector Laboratories, Burlingame, CA) and counterstained with hematoxylin. Images were captured using an Olympus BX43 microscope and Olympus DP73 digital camera (Olympus, Southend-on-Sea, UK).

### Scanning electron microscopy

Skin samples were fixed in 2.5% glutaraldehyde and 4% formaldehyde in 0.1 M HEPES (pH 7.4) at 4 °C for 24 h. Samples were dehydrated through a graded ethanol series and dried in CO_2_ using a Quorum K850 Critical Point Dryer (Quorum Technologies Ltd, Laughton, UK) and gold sputter-coated in a gold–palladium alloy with the Quorum SC7620 Mini Sputter Coater/Glow Discharge System. Samples were imaged using an FEI Quanta 250 scanning electron microscope (ThermoFisher Scientific) to visualise the central point of the wound.

### Toto-1 Iodide and Syto 60 stain

Toto-1 Iodide (2 μM) was applied to excised murine wound surfaces and incubated for 5 min at 37 °C (ThermoFisher Scientific) and counterstained with Syto-60 (10 μM) for 15 min at 37 °C (ThermoFisher Scientific) A Leica TCS SP8 X upright confocal microscope with a HC APO 63x/0.90 W U-V-I lens was used to create Z stack images, 3D reconstruction of Z stacks was performed using Imaris, version 9.3 (Bitplane, Zurich, Switzerland).

### Statistical analyses

Biological and technical replicate data were tabulated and analysed using Graphpad Prism software V9 (GraphPad Software, La Jolla, CA). One way analysis of variance with Dunnets post hoc test for multiple comparisons was utilised to test for differences between each treatment and non-antibacterial control dressings. A *p* value of < 0.05 was considered significant.

## Results

**Figure 1:**
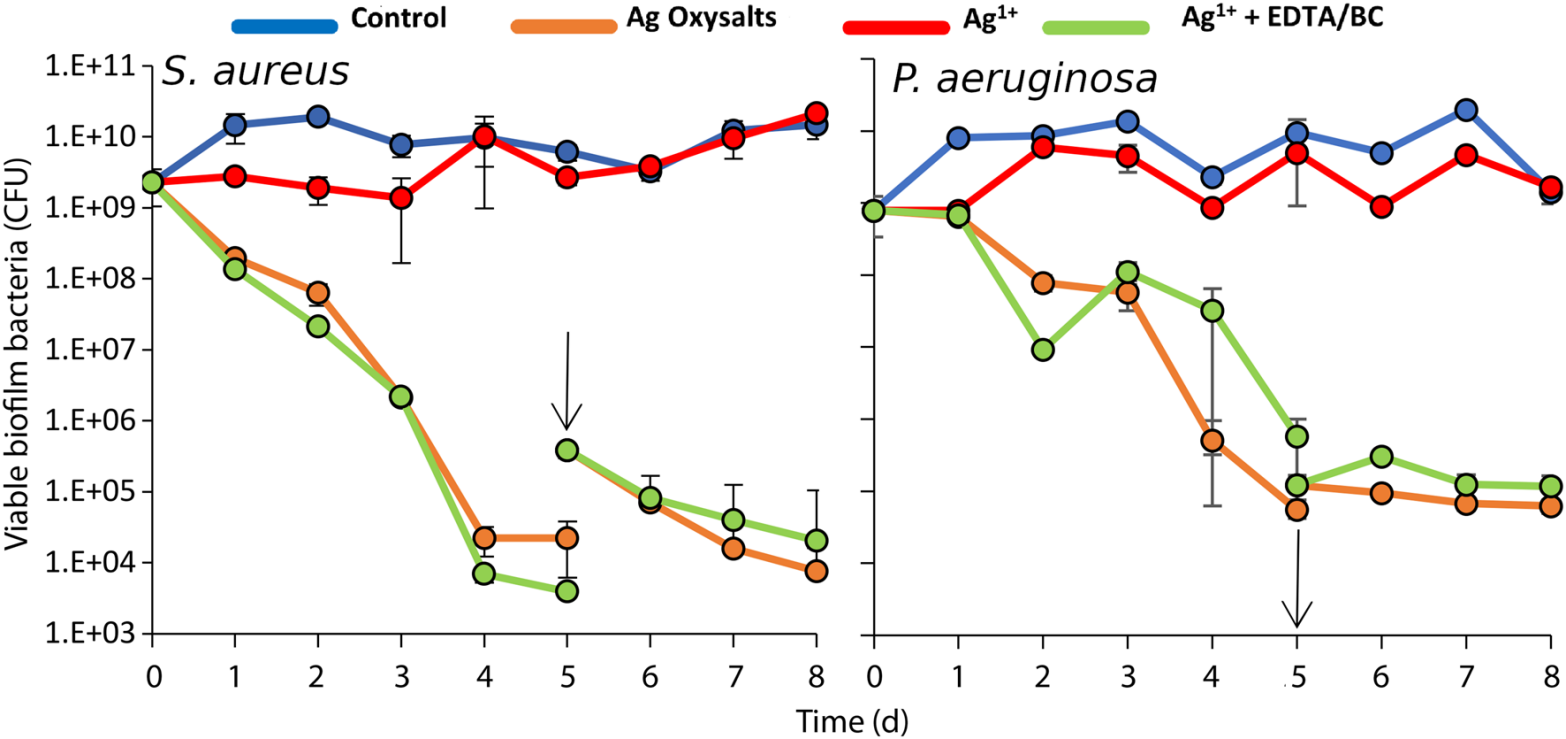
Quantification of viable bacteria in *S. aureus* and *P. aeruginosa* biofilms after treatment with silver dressings. *S. aureus* and *P. aeruginosa* colony biofilms were treated with silver dressings or a non-antimicrobial control dressing and remaining viable bacteria were quantified every 24 h. After 5 days biofilms were re-inoculated with 3.85 × 10^5^ *S. aureus* or 1.22 × 10^5^ *P. aeruginosa* colony forming units of planktonic bacteria respectively, to assess for biofilm reformation. Graph shows mean + /− standard error.

The efficacy of silver gelling fibre dressings was first assessed against *S. aureus* and *P. aeruginosa *in vitro colony biofilms. The silver dressings contained different silver formulations: a traditional silver dressing which produces Ag^1+^ ions; a silver dressing which produces Ag^1+^ ions with the addition of EDTA/BC which is proposed to disrupt the biofilm matrix, exposing the bacteria to the antimicrobial action of silver ions^15^; and a dressing which contains Ag Oxysalts which produces Ag^1+^, Ag^2+^ and Ag^3+^ ions. The efficacy was compared to a non-antimicrobial control gelling fibre dressing. The remaining viable bacteria within the biofilm were assessed every 24 h for 8 days (Fig. 1). At 5 days, biofilms were reinoculated with 3.85 × 10^5^ *S. aureus* or 1.22 × 10^5^ *P. aeruginosa* planktonic bacteria to assess for biofilm reformation. The dressing containing Ag^1+^ exhibited a minimal effect on the viability of bacteria within *S. aureus and P. aeruginosa* biofilms compared to the non-antimicrobial control dressing over 5 days. In contrast, dressings containing Ag Oxysalts and Ag^1+^  + EDTA/BC effectively killed bacteria within the biofilm over 5 days. Reformation of the biofilm was not observed after re-inoculation with planktonic bacteria at day 5 (Fig. 1).

**Figure 2:**
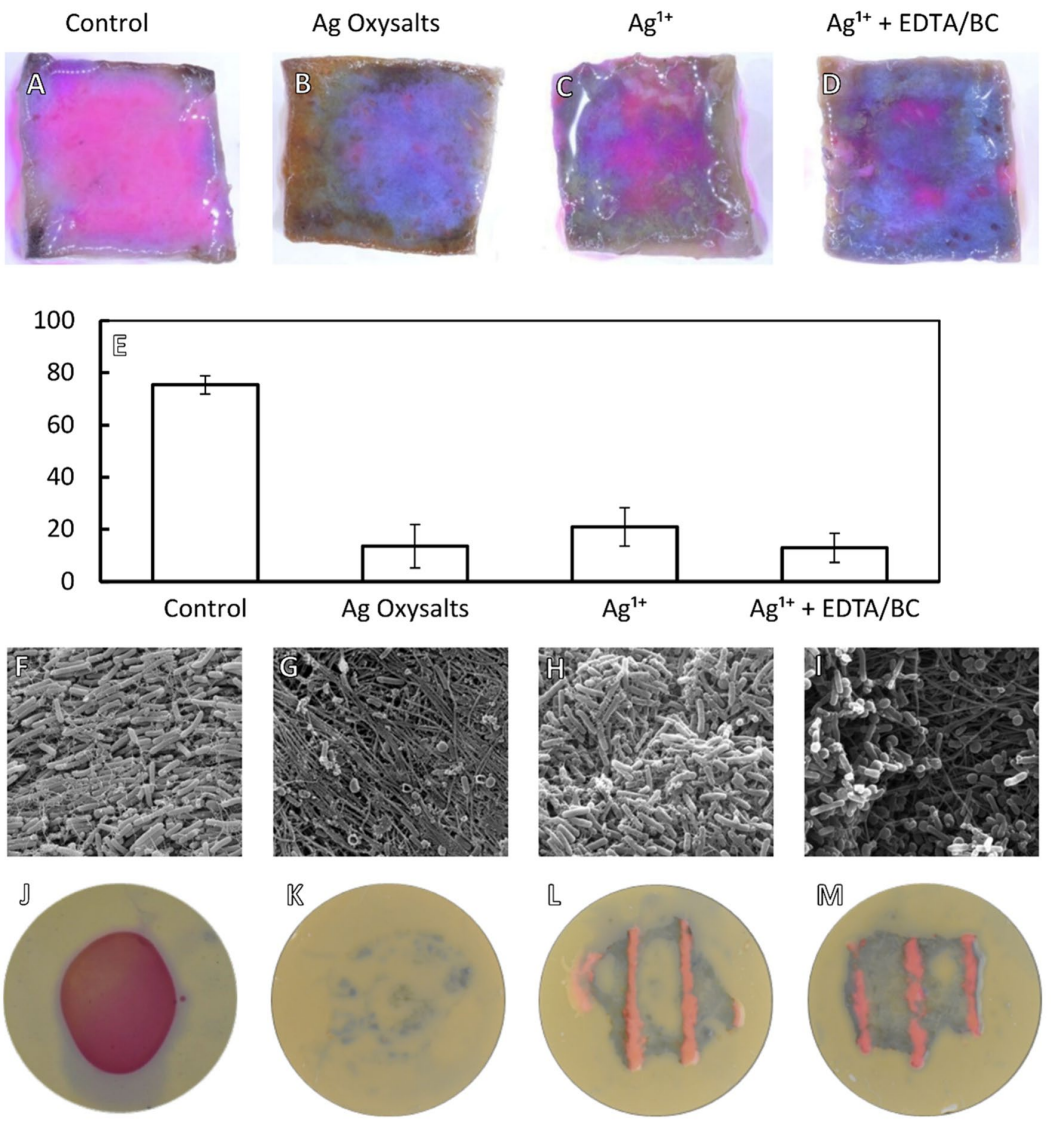
Visualisation of *P. aeruginosa* biofilms after treatment with silver dressings. (**A**–**D**) The viability of bacteria within *P. aeruginosa* biofilms grown on porcine skin were visualised with PrestoBlue viability dye after 24 h treatment with silver dressings or non-antimicrobial control dressings. Viable bacteria appear pink and non-viable bacteria and porcine skin appear blue. (**E**) Viable bacteria (pink staining) were quantified using Image Pro version 10 (**F**–**I**) Scanning electron microscopy of *P. aeruginosa* biofilms grown on porcine skin and treated with silver dressings or non-antimicrobial control dressing for 24 h. SEM scale bar = 5 µm. (**J**–**M**) Colony biofilms grown on filter membranes and stained with PrestoBlue viability dye following 24 h incubation with silver dressings.

To visualise the effects of the silver dressings on biofilm viability, dressings were applied to mature biofilms grown on porcine skin ex vivo. After 24 h, dressings were removed, and the biofilm stained with a blue viability dye which is metabolised to pink by viable bacteria. Biofilms treated with the control dressing appeared pink, indicating viable bacteria within the biofilm (Fig. 2A). In contrast, biofilms treated with Ag Oxysalts dressings appeared predominantly blue indicating the remaining bacteria on the porcine skin surface are non-viable bacteria (Fig. 2B). A mix of blue and pink staining was observed in biofilms treated with dressings containing Ag^1+^, indicating viable and non-viable bacteria within the biofilm (Fig. 2C), whereas the dressing containing Ag^1+^  + EDTA/BC appeared predominantly blue with small patches of pink indicating regions which are not affected by the silver dressing (Fig. 2D). Quantification of viable (pink) and non-viable (blue) regions indicated that the 75% viability for the control dressing (Fig. 2E). The dressings containing Ag^1+^  + EDTA/BC and the Ag Oxysalts dressings performed similarly, with 13% and 14% viability. Ag^1+^ dressings also reduced bacterial viability, with 21% viability. These biofilms were next visualised by scanning electron microscopy (SEM). A layer of *P. aeruginosa* was observed covering the pig skin after treatment with the control dressing and the dressing containing Ag^1+^ (Fig. 2F,H), whereas after treatment with Ag Oxysalts dressings, few bacterial cells were detected, and the underlying collagen fibre of the porcine skin could be visualised (Fig. 2G). Patches of bacteria and patches of underlying collagen fibres could be seen after treatment with the Ag^1+^  + EDTA/BC dressing (Fig. 2I).

To determine if intimate contact between the dressing and biofilm impacts dressing effectiveness, colony biofilms placed on flat surfaces were treated with dressings for 24 h before staining with a viability dye. Untreated biofilms demonstrated a deep-pink staining throughout (Fig. 2J). In contrast to the biofilms treated with dressings containing Ag Oxysalts (Fig. 2K), biofilms treated with dressings containing Ag^1+^ or Ag^1+^  + EDTA/BC exhibited stripes of pink staining (Fig. 2L,M). This pink staining, which indicates viable bacteria, correlated with stitching regions within the dressing. These regions of stitching create dead space allowing the bacteria within the biofilm to survive.

**Figure 3:**
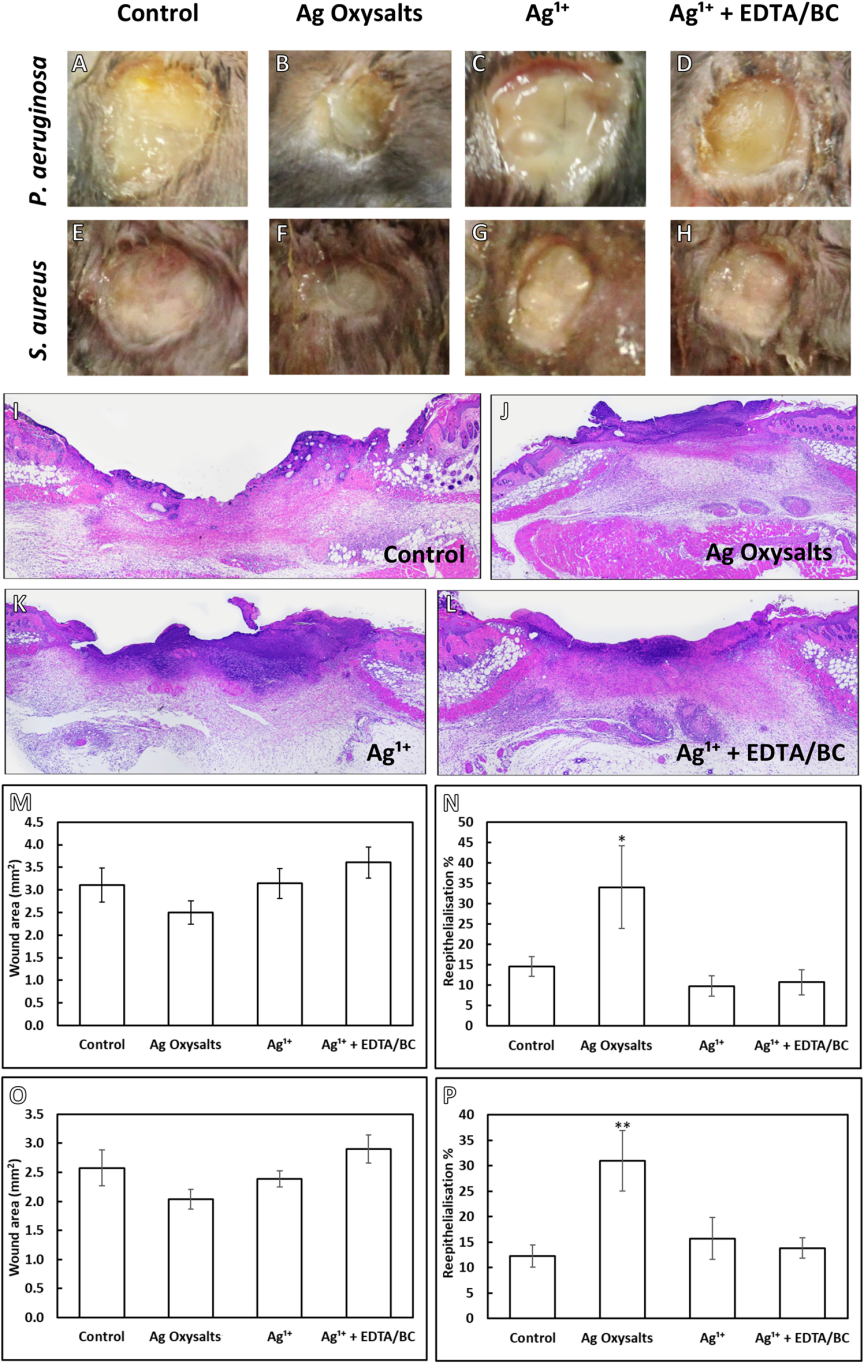
Effects of silver dressings on wound area and re-epithelialisation in biofilm infected wounds. (**A** to **H**) representative macroscopic images of murine wounds infected with *P. aeruginosa* (**A**–**D**) and *S. aureus* (**E**–**H**) biofilms following three days treatment with a non-antibacterial control dressing, Ag Oxysalts dressing, Ag^1+^ dressing and Ag^1+^  + EDTA/BC dressing. (**I**–**L**) Representative *P. aeruginosa* infected, haematoxylin and eosin-stained histological sections used to quantify wound area and reepithelialisation. Quantification of wound area (**M**, **O**) and percentage re- epithelialisation (**N**, **P**) of *P. aeruginosa* (**M**, **N**) and *S. aureus* (**O**, **P**) biofilm infected wounds (n = 12 per treatment group). Graphs show mean + /- standard error. * indicates *p* =  < 0.05; ** indicates *p* =  < 0.01. Macroscopic scale bar = 2.5 mm, histological scale bar = 500 µm.

To assess the effects of silver dressings in vivo, murine, full-thickness, excisional wounds infected with mature *S. aureus* and *P. aeruginosa* biofilms were treated with a non-antimicrobial control or silver dressing. After 3 days of treatment, analysis of macroscopic images highlighted smaller wounds when treated with Ag Oxysalts dressings compared to the non-antimicrobial control dressing and compared to the other silver dressing (Fig. 3A–H). To confirm these observations, wounds were harvested, and wound area and re-epithelialisation quantified from hematoxylin and eosin-stained histological sections using image pro version 10 software (Fig. 3I–L).

Quantification of wound area in *P. aeruginosa* biofilm infected wounds (Fig. 3M) revealed wounds treated with Ag Oxysalts had an average size of 2.5 mm^2^ compared with the non-antibacterial control dressing average wound size of 3.1 mm^2^ which did not reach statistical significance (*p* = 0.423). Wounds treated with Ag^1+^ or Ag^1+^  + EDTA/BC showed no reduction in wound area (3.1mm^2^ and 3.6mm^2^, respectively). Treatment with Ag Oxysalts dressings promoted reepithelialisation (Fig. 3N) to a greater extent than the non-antimicrobial control dressing (34% and 15%, respectively; *p* = 0.029) and Ag^1+^ or Ag^1+^  + EDTA/BC (10% and 11%, respectively).

A similar trend in the wound area and reepithelialisation was also observed in *S. aureus* biofilm-infected wounds (Fig. 3O). Dressings containing Ag Oxysalts reduced wound area (2.0 mm^2^) by 23% compared to the non-antibacterial control dressing (2.6 mm^2^) although this reduction was found to be non-significant (*p* = 0.304) (Fig. 3O). Additionally, a modest reduction in wound area was noted in the Ag^1+^ treated group (2.4mm^2^), and wounds treated with Ag^1+^  + EDTA/BC dressings failing to reduce wound area (2.9mm^2^). Ag Oxysalts also promoted re-epithelialisation of *S. aureus* biofilm-infected wounds (31%), to a greater extent than treatment with the non-antimicrobial control dressing (12%, *p* = 0.003) (Fig. 3P). Ag^1+^ dressings (16%, *p* = 0.903) and Ag^+1^ + EDTA/BC (14%, *p* = 0.965) dressings demonstrated similar levels of reepithelialisation to the control.

**Figure 4:**
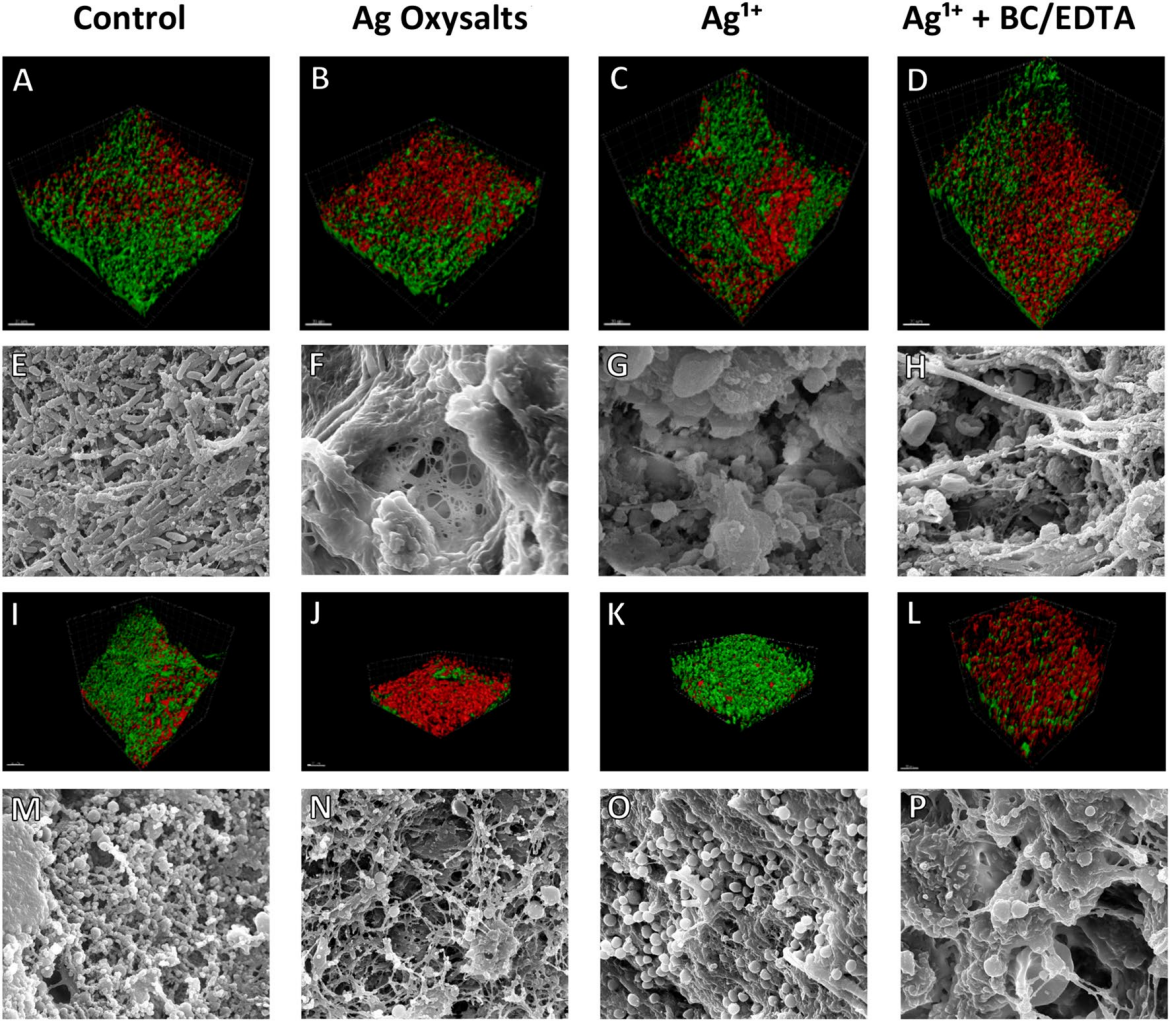
Visualisation of in vivo wound biofilms after 3 days treatment with control or silver dressings. Confocal images of *P. aeruginosa* (**A**–**D**) and *S. aureus* (**I**–**L**) stained with Toto 1 (green) to visualise the extracellular nucleic acids, a component of the biofilm’s extracellular polymeric substance, and Syto 60 (red) which stains intracellular nucleic acids. *P.* Scanning electron microscopy of *P. aeruginosa* (**E**–**H**) & *S. aureus* (**M**–**P**) biofilm infected wounds after 3 days treatment with control and silver dressings. SEM scale bars = 5 µm. Confocal imaging scale bars = 50 µm.

To visualise the effects of the silver dressings on the biofilm matrix, Toto 1 iodide and Syto 60 staining was performed (Fig. 4). Toto 1 iodide is a cell impermeant dye and can be used to accurately visualise extracellular nucleic acids, which are in high quantity in a biofilm EPS. Syto 60 is a cell-permeant dye utilised as a counterstain^16^. Observations of Toto 1 iodide and Syto 60 in *P. aeruginosa* (Fig. 4A–D) and *S. aureus* (Fig. 4I–L) biofilm inoculated wounds revealed a visual reduction in extracellular DNA within the biofilm EPS following 3 days treatment with dressings containing Ag Oxysalts and Ag^1+^  + EDTA/BC. Ag^1+^ dressings which contain no additional anti-biofilm components notably reduced extracellular DNA of *P. aeruginosa* inoculated wounds, however, was less effective in *S. aureus* inoculated wounds.

Scanning electron microscopy revealed a visual reduction in bacteria present within murine wounds inoculated with *P. aeruginosa* (Fig. 4E–H) and *S. aureus* colony biofilms (Fig. 4M–P) when treated for 3 days with all silver dressings.

**Figure 5:**
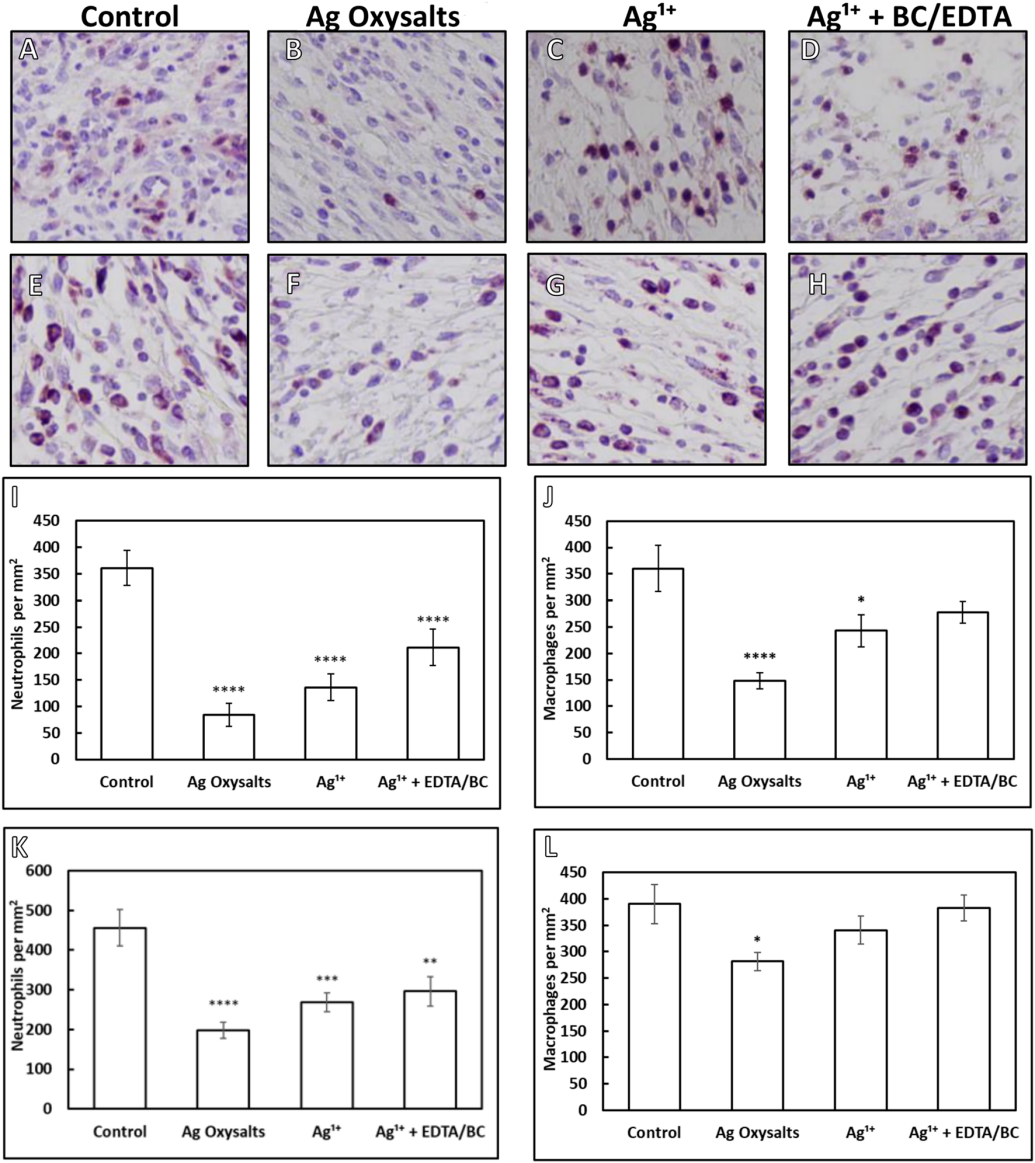
Quantification of neutrophils and macrophages in *P. aeruginosa* and *S. aureus* biofilm infected wounds after 3 days treatment with a non-antimicrobial control or silver dressings. Neutrophils (**A**–**D**) and macrophage (**E**–**H**) were quantified from histological sections stained with neutrophil or macrophage specific antibodies. Quantification of neutrophils (**I** & **K**) and macrophages (**J** & **L**) of *P. aeruginosa* (**I** & **J**) and *S. aureus* (**K** & **L**) biofilm infected wounds. N = 12 per group. Graphs show mean + /− standard error, significance values are in comparison to non-antibacterial control dressing, * indicates *p* =  < 0.05; ** indicates *p* =  < 0.01; *** indicates *p* =  < 0.001; **** indicates *p* =  < 0.0001).

To assess the effects of silver dressings on inflammation in biofilm-infected murine wounds, neutrophils and macrophages within the granulation tissue were quantified from immunohistochemically stained sections of biofilm infected wounds treated for 3 days with control or silver dressings using neutrophil and macrophage-specific antibodies (Fig. 5). All silver dressings reduced the number of neutrophils and macrophages in *P. aeruginosa* infected wounds after three days of treatment compared to the non-antibacterial control dressing. However, treatment with Ag Oxysalts dressings reduced the number of neutrophils (*p* =  < 0.0001) and macrophages (*p* =  < 0.0001) to a greater extent than the other silver dressings tested (Fig. 5I,J). Despite the Ag^1+^  + EDTA/BC having a greater effect on wound biofilms, it reduced neutrophil and macrophage levels to less of an extent than the Ag^1+^ dressing. A similar trend in neutrophil reduction compared to the control was also observed in *S. aureus* biofilm infected wounds after treatment with Ag Oxysalts dressings (*p* =  < 0.0001), Ag^1+^ (*p* = 0.0008) and Ag^1+^  + EDTA/BC (*p* = 0.0043) dressings (Fig. 5K). However, only Ag Oxysalts dressings demonstrated a significant reduction in the number of macrophage within the granulation tissue compared to the control in *S. aureus* biofilm-infected wounds (*p* = 0.0339) (Fig. 5L).

**Figure 6:**
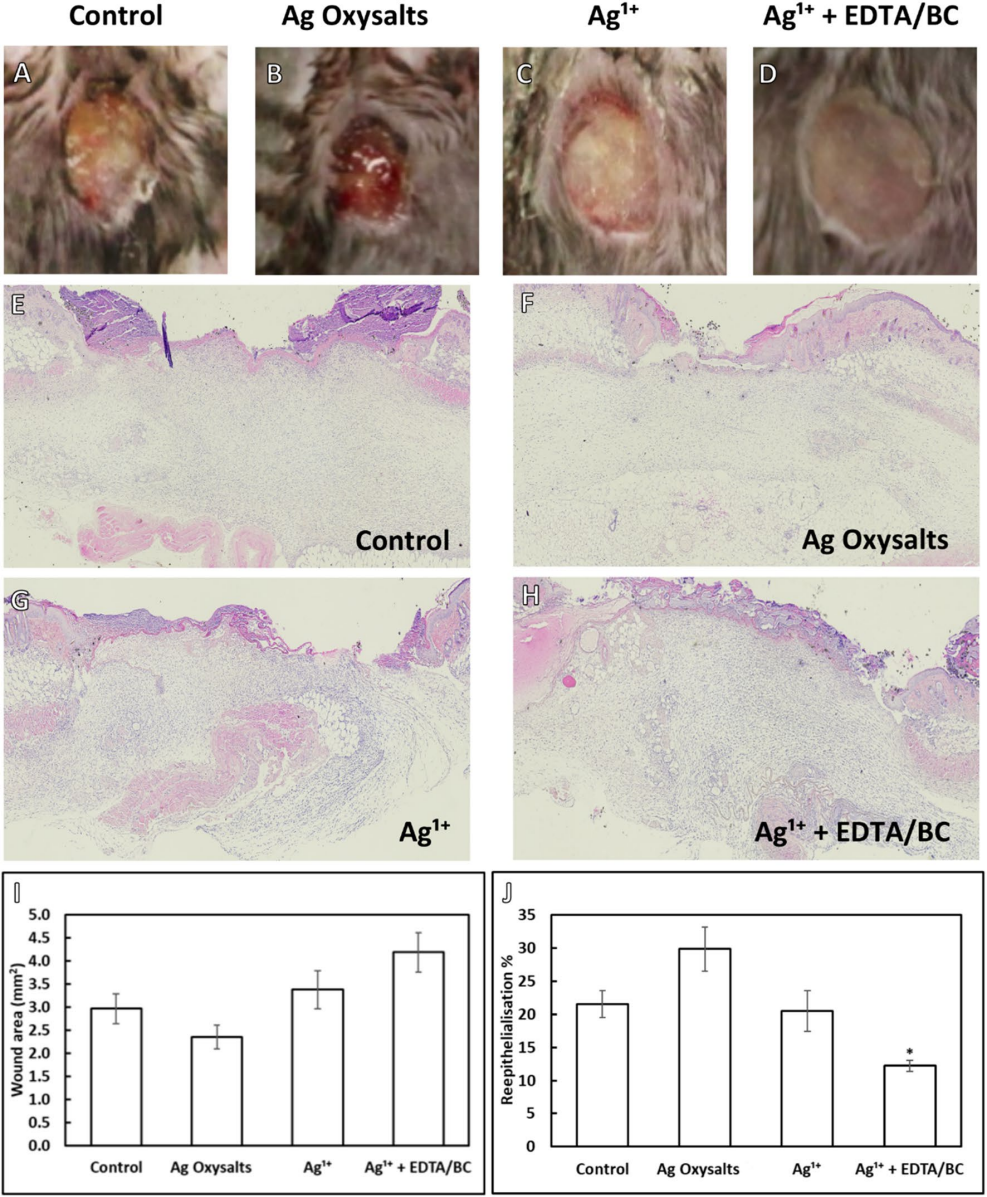
Effects of silver wound dressing on healing of uninfected full-thickness excisional murine wounds. (**A**–**D**) representative macroscopic images of wounds following three days treatment with a non-antibacterial control dressing and silver dressings. (**E**–**H**) Representative haematoxylin and eosin-stained wound sections. Quantification of wound area (**I**) and percentage reepithelialisation (**J**) calculated from histological sections of the wound mid-point using image analysis software (n = 11–12 per treatment group). Graphs show mean + /− standard error. * indicates *p* =  < 0.05.

We next assessed the effects of the silver dressings on healing independent of infection. Uninfected excisional wounds were treated for 3 days with a non-antimicrobial control dressing or silver dressing (Fig. 6). Of the silver dressings tested, only wounds treated with Ag Oxysalts dressings appeared smaller compared to control-treated wounds from macroscopic images (Fig. 6A–D). Quantification of wound area from histological analysis revealed treatment with Ag Oxysalts dressings resulted in an average wound area of 2.35 mm^2^ compared to 2.96 mm^2^ in control-treated wounds, however this difference did not reach statistical significance (*p* = 0.488) (Fig. 6I). In contrast, no reduction in wound area compared to the control was observed after treatment with Ag^1+^ (3.38 mm^2^, *p* = 0.757) or Ag^1+^  + EDTA/BC (4.18 mm^2^, *p* = 0.054) dressings. Increased re-epithelialisation was observed with Ag Oxysalts dressings compared to the control (30% vs 22%, respectively), although this did not achieve significance (*p* = 0.067), it is highly suggestive and supports previous findings that Ag Oxysalts dressings promote re-epithelialisation of uninfected wounds^17^. In contrast, treatment with Ag^1+^ or Ag^1+^  + EDTA/BC dressings had no effect or exhibited a reduction in reepithelialisation compared to the control.

## Discussion

Silver has a long-standing history of use as an antimicrobial therapy for wound care, however the many different formulations and methods of delivery may account for variations in antimicrobial efficacy^18^. Furthermore, the antibiofilm properties of specific silver delivery systems are incompletely understood. Whilst the host immune response is comparatively effective at combatting planktonic bacteria, it is generally less effective against biofilms^19^. Planktonic bacteria can be readily phagocytosed by macrophages, but within a biofilm, aggregated cells present more of a challenge, limiting the host response to the point where immune cells may undergo apoptosis releasing proinflammatory factors to heighten the immune response^20^. It has been observed that some leukocytes can penetrate biofilms^21^ but may be unable to engulf the bacteria upon breaching this defence^22^. To support the host immune response in combatting wound biofilm infections, a holistic approach should be undertaken. Debridement of the wound can physically disrupt the biofilm, removing the bulk of the bioburden, but remaining pockets of biofilm may not be effectively combatted by the host immune response, especially if the host immune response is compromised. Therefore, antimicrobial therapies such as silver dressings can support the host immune response in eliminating the biofilm infection. The formulation, concentration, solubility and delivery substrate can affect the antimicrobial efficacy of silver. In recent years, there have been advances in silver dressing technology to make these dressings more potent^9,23^. As silver dressing technology advances, it is important to understand the effectiveness of these dressings against wound infections and, importantly the effect that these potent forms of silver have on the wound environment and healing.

In this study, we compared the effectiveness of two advanced silver dressings with a traditional silver dressing that produces Ag^1+^ ions against biofilms using a variety of in vitro and in vivo models. We also assessed the effects of these dressings on the wound environment and healing independent of infection. To minimise any effect from the delivery substrate all silver dressing tested were composed of carboxymethylcellulose.

Our initial assessment of these silver dressings against *P. aeruginosa* and *S. aureus* colony biofilms showed that unlike the traditional Ag^1+^ dressing, the two advanced silver dressings, Ag^1+^  + EDTA/BC and Ag Oxysalts, effectively killed bacteria within a biofilm over 5 days. Furthermore, when rechallenged with planktonic bacteria, these dressings prevented biofilm reformation. The Ag^1+^ dressing, which contains silver chloride, the same silver compound and base substrate as the Ag^1+^  + EDTA/BC showed limited effect on the viability of bacteria within a biofilm over the same period. The observation that the Ag^1+^  + EDTA/BC dressing is more effective at combatting biofilm than the Ag^1+^ dressing composed of the same substrate and silver compound supports the claim that additional components are required to enhance the efficacy of silver chloride against biofilm and has been reported elsewhere^15^. These findings support the concept that BC and EDTA have additional effects which contribute to the overall efficacy of the dressing and that an absence of this component in the Ag^1+^ dressing can lead to a failure to demonstrate efficacy in vitro. We found that Ag Oxysalts dressings which produce Ag^2+^ and Ag^3+^ ions exhibit greater antimicrobial efficacy than Ag^1+^ and to a similar level as Ag^1+^  + EDTA/BC. However, due to its high redox potential, it is unclear how long Ag^3+^ ions remain active to be effective against wound biofilms and thus merits further investigative studies. Furthermore, there are many different dressings that generate Ag^1+^ ions that were not tested in this study, these dressings are composed of different silver compounds, concentrations, and base substrate which may affect the delivery of Ag^1+^ ions and effectiveness against biofilms. It is also worth noting that there are many different in vitro and in vivo models used to assess the effectiveness of wound dressings on biofilms. The type of model used, and the salt and protein content of the medium used in these models can affect the efficacy of the dressings. In our in vivo model, we allowed biofilms to mature in vitro before transferring them to the dermal surface of the wound. The murine host immune response is relative effective at combatting planktonic bacteria applied to a wound and therefore formation of a biofilm occurs as the wound is healing. Adding a mature biofilm to the wound limits the effectiveness of the host immune response on biofilm formation, allowing a mature biofilm to establish within the wound before healing commences. Thus, our model enables the effectiveness of antimicrobial dressings on a mature biofilm to be assessed before the wound has begun to heal.

We also found that the conformability of the dressing affected the efficacy of the silver dressings against biofilms grown in vitro and on porcine skin. Intimate contact with the wound is thought to be critical to the antimicrobial efficacy of a dressing^24,25^. The dressing containing Ag Oxysalts made intimate contact with mature biofilms resulting in the visible reduction of viable bacteria within the biofilm after 24 h. In contrast, significant areas of viable bacteria remained when treated with Ag^1+^ and Ag^1+^  + EDTA/BC dressings. These dressings contain stitching throughout the dressing, which creates dead space that prevents intimate contact with the biofilm. In our in vitro study, these non-contact areas impeded the eradication of viable bacteria within the biofilm. We only assessed the viability of bacteria after 24 h treatment and it is likely that over time as the dressing becomes more saturated, there will be less dead space which will reduce these regions of viable bacteria. However, this highlights the importance of the dressings’ constitution and not just the type of silver within the dressing.

While in vitro investigations are useful to compare the efficacy of different silver technologies, it is important to understand the impact of these dressings on biofilms in vivo, where the host tissue and immune response contributes to the effectiveness of the dressings against biofilm. The impact of these dressings on the wound biofilm was observed by scanning electron microscopy and via staining the biofilms’ EPS using intracellular and extracellular DNA dyes. We found that all dressings were effective at reducing the extracellular DNA of biofilm-infected wounds after 3 days treatment, although the Ag^1+^ dressing was less effective against *S. aureus*-infected wounds. Scanning electron microscopy also revealed a visible reduction in bacteria present within the wounds treated with the silver dressings, although this was more pronounced with the Ag Oxysalts dressings and Ag^1+^  + EDTA/BC dressings compared to the Ag^1+^ dressing. This data highlights that the silver dressings tested impact the structure of the biofilm to different extents; however, none of the silver dressings eradicated the biofilm and therefore it confirms the need for a holistic approach in treating wound biofilm infections, such as physical debridement to remove the bulk of the biofilm prior to treatment with silver dressings.

Chronic wounds often exist in a heightened state of inflammation, whereby excess inflammatory cells reside within the wound tissue for prolonged periods, causing tissue damage and depleting the wound of oxygen essential for effective cellular metabolism and function^26^. Biofilms can exacerbate this hostile wound environment, negatively impacting healing in several ways including, inhibiting cell proliferation and migration, and upregulating proinflammatory cytokines^27^. As silver dressings become more potent it is important to understand the impact of these dressings on the wound environment and healing.

Interestingly, despite the impact of all silver dressings on the biofilm composition, only the Ag Oxysalts dressings increased the re-epithelialisation of these infected wounds. This data supports our previous findings^17^ and that of Kalan et al*.* (2017)^28^ who demonstrated that Ag Oxysalts have a good safety and toxicity profile due to lower concentrations of silver being effective against biofilms.

Our current study highlights the difference in silver technology between antimicrobial silver dressings and the impact the technology has on the wound environment and healing independent of infection. These findings, however, differ to previous findings in which Ag^1+^  + EDTA/BC dressings demonstrated an improvement in healing parameters in in vivo punch wounded rabbit ears. However this may be due to differences in the animal model, measurement time points and method of bacterial application^29^. Wound measurements, in this case, were taken 12 days post wounding allowing for the active ingredients of the dressing to act upon a biofilm over a longer period. This is supported by a study which demonstrated that clinically infected leg ulcers treated with Ag^1+^  + EDTA/BC initially increased in size after one week of treatment, before reducing ulcer area over the following 3 weeks of Ag^1+^  + EDTA/BC treatment followed by 4 weeks treatment with a non-antibacterial CMC dressing^30^.

Certain forms and concentrations of silver have previously been shown to have a cytotoxic effect in vitro^11^, however, these in vitro findings do not always translate to an adverse effect in vivo. Furthermore, the evolution of silver technology and a greater understanding of silver compounds and concentration within the dressing has led to the development of many safe and effective silver dressings. However, as silver dressing technology advances, it is important to understand the impact of these dressings on the wound environment^31–33^. Increases in the rate of reepithelialisation have previously been reported to correspond with an increase in the ratio of anti-inflammatory M2 macrophages compared to pro inflammatory M1 phenotype. This was noted in a previous murine model when a silver hydrogel wound dressing was compared with silver sulfadiazine and non-antimicrobial hydrogel^34^.

Chronic wounds can exhibit excessive amounts of inflammation, and it has been observed that the presence of excessive neutrophils can be detrimental to wound repair^35^. In a study of neutrophil-depleted mice^36^, the presence of neutrophils delayed re-epithelialisation. The presence of excessive quantities of neutrophils gives rise to high levels of proteases and reactive oxygen species such as superoxide and hydrogen peroxide which have been implicated in chronic and slow-to-heal wounds^37,38^. Similarly, increased macrophage quantities, if left unchecked, can contribute to delayed wound healing^39^. This increase is particularly relevant if macrophages then fail to progress from a pro-inflammatory to a pro-healing phenotype, leaving a wound unable to proceed out of the inflammatory phase of healing^40^. We observed a reduction in neutrophil and macrophage numbers within the biofilm-infected wounds after 3 days treatment with all the silver dressings, although the reduction was more pronounced with the Ag Oxysalts dressings. This reduction may occur as a direct result of the silver on the immune response, in response to a reduction in bioburden or due to the wounds being at a more advanced stage of healing and thus the immune cells within the wound are diminishing. This reduction in inflammatory cells within the wound is likely to support a favourable wound environment for healing. The mechanism of action to how Ag Oxysalts promote healing independent of infection remains unclear, however the ability of Ag Oxysalts to produce oxygen and also to breakdown detrimental levels of the inflammatory mediator, hydrogen peroxide may explain and requires further studies^17^.

Chronic non-healing infected wounds are a challenge for clinicians and patients alike. While many dressings have claims of antimicrobial efficacy, studies rarely focus on other crucial factors contributing to the wound microenvironment. This study revealed different silver technologies have different antimicrobial efficacies and importantly have different impacts on the wound environment and healing independent of infection. Whilst these in vitro and in vivo studies shed light on the effectiveness of these dressings to treat wound infection and improve healing, randomised controlled trials are required to assess the effectiveness of these dressings within the clinic.

## Data Availability

The datasets used and/or analysed during the current study are available from the corresponding authors on reasonable request.
